# Exploring the association between moral injury and posttraumatic stress symptoms among Canadian public safety personnel

**DOI:** 10.1002/jts.23122

**Published:** 2024-12-16

**Authors:** Andrea M. D'Alessandro‐Lowe, Andrew M. Scott, Herry Patel, Bethany Easterbrook, Kimberly Ritchie, Andrea Brown, Mina Pichtikova, Mauda Karram, Emily Sullo, James Mirabelli, Hygge Schielke, Ann Malain, Charlene O'Connor, Shannon Remers, Ruth Lanius, Randi E. McCabe, Margaret C. McKinnon

**Affiliations:** ^1^ Department of Psychology, Neuroscience & Behaviour McMaster University Hamilton Ontario Canada; ^2^ Homewood Research Institute Guelph Ontario Canada; ^3^ St. Joseph's Healthcare Hamilton Hamilton Ontario Canada; ^4^ Department of Psychiatry University of California San Diego La Jolla California USA; ^5^ Trent/Fleming School of Nursing Trent University Peterborough Ontario Canada; ^6^ Department of Psychiatry and Behavioural Neurosciences McMaster University Hamilton Ontario Canada; ^7^ Department of Applied Psychology and Human Development University of Toronto Toronto Ontario Canada; ^8^ Lawrence Bloomberg Faculty of Nursing Univerity of Toronto Toronto Ontario Canada; ^9^ Homewood Health Centre Guelph Ontario Canada; ^10^ Department of Psychiatry University of Western Ontario London Ontario Canada

## Abstract

Public safety personnel (PSP), such as police officers, firefighters, correctional workers, and paramedics, routinely face work stressors that increase their risk of developing posttraumatic stress disorder (PTSD). PSP may additionally face moral transgressions in the workplace (e.g., witnessing human suffering, working within broken systems), heightening the risk of moral injury (MI) in this population. Research among military personnel and health care workers shows an association between MI and PTSD; however, less is known about the association between these constructs among PSP. Canadian PSP completed an online survey between June 2022 and June 2023, including a demographic questionnaire and measures of PTSD, MI, dissociation, depression, anxiety, stress, and childhood adversity. Latent variable structural equation modeling (SEM) was performed to ascertain the impact of a latent MI construct (i.e., shame, trust violation, functional impairment) on a latent PTSD construct (i.e., intrusions, avoidance, negative alterations in cognition and mood, hyperreactivity, depersonalization, derealization). Sex, age, depression, anxiety, stress, and childhood adversity were included as covariates. A total of 314 PSP were included in the data analysis. A latent variable SEM regressing PTSD onto MI and including covariates accounted for 83.7% of the variance in PTSD. MI was the strongest predictor compared to all covariates and was significantly associated with PTSD symptoms, β = .506, *p* < .001, above and beyond the impacts of sex, age, depression, anxiety, stress, and childhood adversity. These findings are consistent with research among military members and health care providers and highlight the importance of further exploring MI among PSP.

Public safety personnel, including police officers, firefighters, correctional workers, paramedics, dispatchers, and other public safety personnel (PSP), appear to be at an elevated risk for mental health disorders, including posttraumatic stress disorder (PTSD), relative to the general population (Bahji et al., 2022; Carleton et al., 2019, 2018; Lentz et al., 2021). Indeed, almost half of the 5,013 Canadian PSP surveyed in a 2016 study screened positive for at least one mental health condition (e.g., PTSD, major depressive disorder [MDD], panic disorder, generalized anxiety disorder [GAD], alcohol use disorder [AUD]; Carleton et al., 2018); comparatively, positive screens for mental health conditions were approximately 10% in the general Canadian population in 2012 (Statistics Canada, 2012).

Elevated rates of PTSD and other mental health conditions among PSP may partly be related to frequent and repeated exposure to potentially traumatic events (PTEs), including actual or threatened death, serious injury, or sexual violence (American Psychiatric Association [APA], 2022; Heber et al., 2023), in the workplace. For example, killing or seriously injuring someone in the line of duty was found to be significantly associated with PTSD in a sample of 400 police officers (Komarovskaya et al., 2011). Similarly, virtually all participants in a sample of approximately 4,000 PSP endorsed exposure to violent or accidental death, serious transportation accidents, and assaults, with PSP typically endorsing exposure to at least 11 instances of each event (Carleton et al., 2019). Whereas Carleton et al. (2018) found that approximately one in four of the 5,013 PSP in their sample who experienced a PTE screened positive for PTSD, approximately 5%–10% of individuals in the general population who experience a PTE go on to develop symptoms consistent with a diagnosis of PTSD (Ozer et al., 2003).

According to the criteria outlined in the *Diagnostic and Statistical Manual of Mental Disorders* (5th ed.; *DSM‐5*; APA, 2013), PTSD is a stressor‐related disorder diagnosed in individuals who have experienced a Criterion A traumatic event (i.e., PTE) and demonstrate prolonged intrusion symptoms (e.g., flashbacks, nightmares), avoidance behaviors (e.g., avoiding people or places associated with the event), negative alterations in cognition or mood (i.e., negative beliefs about the self and others), and alterations in arousal and reactivity (e.g., hypervigilance. PTSD is associated with significant cognitive, emotional, and functional impairment (Alves de Araujo Junior et al., 2023; Boyd et al., 2019; Jellestad et al., 2021); substance use (Patel et al., 2021, 2022, 2023); suicidality, and other mental health difficulties (Panagioti et al., 2015; Ramsawh et al., 2014). The *DSM‐5* acknowledged heterogeneity within PTSD by introducing the dissociative subtype (APA, 2013)—individuals diagnosed with PTSD who experience persistent or recurrent depersonalization (e.g., detachment from one's mental processes and body) or derealization (e.g., the sense that one's surroundings are unreal or distant) may be diagnosed with this subtype. This addition to the *DSM‐5* is consistent with neurobiological research on distinct profiles of individuals with a prototypical presentation of PTSD and those with the dissociative subtype (Lanius et al., 2012, 2010; Shaw et al., 2022). It is estimated that up to 40% of individuals who are diagnosed with PTSD may experience persistent or recurrent depersonalization or derealization (White et al., 2022), demonstrating the relevance of the addition of the dissociative subtype into traumatic stress nosology.

Recent evidence suggests that moral injury (MI) may also be relevant among PSP (Easterbrook et al., 2022; Lentz et al., 2021; Lloyd et al., 2021; Phelps et al., 2023; Roth et al., 2022, 2023; Smith‐Macdonald et al., 2021). MI is the psychological, social, spiritual, and behavioral distress and impairment an individual experiences after their moral values are violated by themselves or others, namely potentially morally injurious events (PMIEs; Jinkerson, 2016; Litz et al., 2009). MI may involve intense moral emotions, such as shame, guilt, anger, or betrayal, that can impact self‐perception, socialization, and beliefs about one's meaning and purpose and lead to self‐harming or sabotaging behavior (Litz et al., 2022). Litz et al. (2022) distinguished between shame‐related outcomes (e.g., “I am not the good person I thought I was”), which typically stem from self‐perpetrated PMIEs, and trust violation–related outcomes (e.g., “I have lost faith in humanity”), which emerge following PMIEs perpetrated by others. MI is not presently a diagnosable mental health disorder but has been linked to various mental health disorders, including PTSD and MDD (Easterbrook et al., 2023; Levi‐Belz et al., 2020; Maguen et al., 2022; Phelps et al., 2024; Protopopescu et al., 2021; Wisco et al., 2017). Although MI remains understudied among PSP compared with other service groups (e.g., military personnel, health care workers), it appears to be a relevant construct among PSP (Easterbrook et al., 2022; Lade et al., 2023; Lentz et al., 2021; Phelps et al., 2023; Rodrigues et al., 2023; Smith‐Macdonald et al., 2021). Indeed, PSP endorse a range of PMIEs, including having to make life‐and‐death decisions, witnessing human suffering, working within impaired social systems, and betrayal from leaders. Furthermore, these experiences have been connected to symptoms of PTSD, guilt, shame, and betrayal among PSP.

MI and PTSD have oft been found to present in tandem (Barnes et al., 2019; Benfer et al., 2023; Koenig et al., 2020; Litz et al., 2018; Maguen et al., 2022). Indeed, Koenig et al. (2020) explored the association between MI and PTSD among U.S. veterans and military personnel and found that MI was related to all *DSM‐5* PTSD symptom clusters. In a sample of treatment‐seeking U.S. military personnel, Litz et al. (2018) found that individuals who reported MI related to self‐perpetrated events endorsed higher levels of reexperiencing (i.e., intrusions) symptoms, guilt, and self‐blame relative to those who reported life‐threat trauma that involved self‐perpetration. Finally, Benfer et al. (2023) used network analysis to explore the associations among MI, PTSD, and depression in military personnel and found that these three outcomes appeared to be interconnected by way of PTSD‐related negative alterations in cognition and mood and moral injury–related functional impairment. This co‐occurrence may relate to the similar features of PTEs and PMIEs, which give rise to PTSD and MI, respectively, as both types of stressful events may include death, harm, or moral violations (Carleton et al., 2019; Phelps et al., 2023). Indeed, PMIE exposure has been associated with increased odds of a past‐year PTSD diagnosis in Canadian military personnel and veterans (Easterbrook et al., 2023). Moreover, MI and PTSD may share several features, such as intrusions, avoidance, shame, anger, changes in beliefs about one's self and others, social withdrawal, self‐destructive behavior, and spiritual or existential crises (Barnes et al., 2019; Benfer et al., 2023; Litz et al., 2009). Several authors, however, have suggested that the underlying motivation behind these processes and behaviors may differ such that these symptoms may be more closely related to fear in PTSD and shame or guilt in MI (Benfer et al., 2023; Currier et al., 2018; Litz et al., 2018, 2009).

D'Alessandro‐Lowe et al. (2024) explored the association between MI and PTSD symptoms, including dissociative symptoms, in a sample of Canadian health care workers. The study identified a latent MI construct, which included both shame and trust violation–related morally injurious outcomes, functional impairment, and team‐ and system‐level moral distress, as significantly and positively associated with a latent PTSD construct (i.e., intrusions, avoidance, negative alterations in cognition and mood, heightened reactivity, and depersonalization and derealization) when controlling for the effects of age, sex, depression, anxiety, stress, and childhood adversity (β = .813, *p* < .001).

Despite emerging evidence, research investigating the association between MI and PTSD in PSP is limited. Accordingly, the purpose of the present study was to replicate D'Alessandro‐Lowe et al.’s (2024) work among health care workers and explore the association between MI and PTSD, including symptoms of dissociation, among a sample of Canadian PSP. Sex, age, depression, anxiety, stress, and childhood adversity were included in the model as covariates based on associations between these variables and PTSD (Frewen et al., 2019; Price & Van Stolk‐Cooke, 2015; Yehuda et al., 2001) or MI (Battaglia et al., 2019; Protopopescu et al., 2021; Roth et al., 2022). Specifically, we sought to understand the strength of the association between MI and PTSD above and beyond the impacts of sex, age, depression, anxiety, stress, and childhood adversity on PTSD. We expected to find results similar to those reported by D'Alessandro‐Lowe et al. (2024) given that health care workers and PSP face increased exposure to PTEs and PMIEs in their lines of work (Carleton et al., 2019; D'ettorre et al., 2021). We hypothesized that the full model would account for a significant proportion of the variance in the PTSD latent variable and that the MI latent variable would be significantly and positively associated with the PTSD latent variable.

## METHOD

### Participants and procedure

A total of 314 participants were included in the data analyses (Table 1). The average participant age was 41.90 years (*SD* = 11.10, range: 18–72 years). On average, participants had worked in public safety for 14.20 years (*SD* = 9.90, range: 0–45 years). Descriptive statistics for self‐report questionnaire scores are presented in Table 2.

**TABLE 1:** Sociodemographic characteristics of the sample

Variable	*n*	%
Sex		
Male	220	70.1
Female	94	29.9
Gender		
Man	218	69.4
Woman	94	29.9
Other	< 5	
Missing	1	0.3
Ethnicity^a^		
African	< 5	
Caribbean	5	1.6
East Asian	< 5	
Indigenous	25	8.0
Latin American	< 5	
Pacific Islander	< 5	
South Asian	< 5	
Southeast Asian	< 5	
European	219	69.7
Other	45	14.3
Province		
British Columbia	29	9.2
Alberta	69	22.0
Saskatchewan	15	4.8
Manitoba	10	3.2
Ontario	146	46.2
Quebec	7	2.2
New Brunswick	< 5	
Nova Scotia	21	6.7
Prince Edward Island	< 5	
Newfoundland/ Labrador	8	2.5
Northwest Territories	< 5	
Yukon	< 5	
Profession		
Firefighter	53	16.9
Police officer	52	16.6
Correctional worker	67	21.3
Paramedic	106	33.8
Border patrol	< 5	
Dispatcher	8	2.5
Other	23	7.3
Missing	1	0.30

*Note*: When fewer than five participants endorsed a given characteristic, results were suppressed to protect anonymity.

^a^ Participants were permitted to select multiple options.

**TABLE 2:** Descriptive statistics for constructs of interest

Construct	Measure	Possible range	*M*	*SD*	Cronbach's α
Moral injury					
Shame	MIOS	0–28	9.86	6.42	.86
Trust violation	MIOS	0–28	14.45	5.83	.80
Functional impairment	MIOS	0–6	3.13	1.93	‐
PTSD symptoms (PTSS)^a^					
Intrusions	PCL‐5	0–20	7.70	5.43	.92
Avoidance	PCL‐5	0–8	3.63	2.64	.89
NACM	PCL‐5	0–28	11.32	7.19	.89
Alterations in arousal and reactivity	PCL‐5	0–24	10.77	6.16	.87
Total	PCL‐5	0–80	33.42	19.15	‐
Dissociation					
Depersonalization	MDI	5–25	8.98	3.83	.79
Derealization	MDI	5–25	9.33	3.88	.84
Depressive symptoms	DASS‐21^b^	0–42	15.49	10.24	.91
Anxiety symptoms	DASS‐21^b^	0–42	11.97	8.65	.85
Stress‐related symptoms	DASS‐21^b^	0–42	17.91	8.91	.87
Childhood adversity	ACES	0–10	2.76	2.33	‐

*Note*: MIOS = Moral Injury Outcomes Scale; PTSD = posttraumatic stress disorder; PTSS = posttraumatic stress symptoms; PCL‐5 = PTSD Checklist for *DSM‐5*; NACM = negative alterations in cognitions and mood; MDI = Multiscale Dissociation Inventory; DASS‐21 = Depression Anxiety Stress Scale–21.

^a^ Total PCL‐5 scores equal to and above 33 indicate potential PTSD.

^b^ Clinical cutoffs are as follows: Depression subscale: normal (0–9), mild (10–13), moderate (14–20), severe (21–27), extremely severe (≥ 28); Anxiety subscale: normal (0–7), mild (8–9), moderate (10–14), severe (15–19), extremely severe (≥ 20); Stress subscale: normal (0–14), mild (15–18), moderate (19–25), severe (26–33), extremely severe (≥ 34).

Data were drawn from a larger dataset of survey responses from Canadian PSP. Hamilton Integrated Research Ethics Board (#12667) granted permission for this study. Portions of this dataset have been used in research on PSP substance use during the COVID‐19 pandemic (Patel et al., 2023) and the associations between MI and coping strategies among PSP during the COVID‐19 pandemic (D'Alessandro‐Lowe et al., in press). The survey was housed on REDCap (Harris et al., 2019, 2009). PSP were recruited between June 2022 and June 2023 via social media posts and emailed letters. Eligible PSP were at least 18 years old, able to speak and read English, living in Canada, and employed as PSP. All participants provided electronic informed consent before accessing the survey, and all participants included in this analysis consented to secondary analysis of their data.

### Measures

#### Demographic characteristics

Participants filled out a demographic questionnaire that queried age, sex, gender, province or territory of residence, occupation, and years worked.

#### MI

The Moral Injury Outcomes Scale (MIOS; Litz et al., 2022) was used to assess MI. The primary MIOS items have a two‐factor structure, including shame‐related (e.g., “I am not the good person I thought I was”) and trust violation–related outcomes (e.g., “People don't deserve second chances”). Participants were asked to use their self‐identified “worst” PMIE as a reference and rate their degree of agreement with 14 items on a scale of 0 (*strongly disagree*) to 4 (*strongly agree*), with higher scores indicating greater MI. A single MIOS item assessing functional impairment, rated on a scale of 0 to 6, was also used in the present analysis. The MIOS has demonstrated sound psychometric properties and has been shown to be a valid and reliable tool among military service members (Litz et al., 2022) and acute care nurses (Tao et al., 2023). In the present sample, Cronbach's alpha was .86 for the MIOS Shame subscale and .80 for the Trust Violation subscale.

#### PTSD symptoms

The 20‐item PTSD Checklist for DSM‐5 (PCL‐5; Weathers et al., 2013) was used to assess symptoms of PTSD, including intrusions, avoidance, negative alterations in cognition and mood, and alterations in arousal and reactivity. Participants were asked to rate their degree of agreement with each item based on their past‐month experience on a scale of 0 (*not at all*) to 4 (*extremely*), with higher scores indicating higher symptom levels. The PCL‐5 previously demonstrated strong internal consistency, convergent validity, and discriminant validity in a sample of first responders (Morrison et al., 2021). In the present sample, Cronbach's alpha values for each symptom cluster–specific subscale ranged from .87 to .92.

#### Depersonalization and derealization

The Depersonalization and Derealization subscales of the Multiscale Dissociation Inventory (MDI; Briere et al., 2005) were used to assess constructs relevant to the PTSD dissociation subtype and included in the PTSD latent variable construction. Participants were asked to indicate the degree to which they had experienced symptoms of depersonalization and derealization over the last month, scoring their responses on a scale of 1 (*never*) to 5 (*very often*), with higher scores indicating higher levels of a given construct. The MDI has demonstrated strong internal consistency and convergent and divergent validity among adults and undergraduate students (Jeffirs et al., 2023). In the present sample, Cronbach's alpha values were .79 and .84 for the MDI Depersonalization and Derealization subscales, respectively.

#### Depression, anxiety, and stress

The Depression Anxiety Stress Scales–21 (DASS‐21; Lovibond & Lovibond, 1995) were used to assess depression, anxiety, and stress symptoms. Participants were asked to rate their degree of agreement with each item on a scale of 0 (*never*) to 3 (*almost always*), using their past‐week experience as a reference point. Scores are summed and multiplied by two for each subscale, with higher scores indicating higher symptom levels. The DASS‐21 has demonstrated satisfactory psychometric properties, including validity and reliability (Lovibond & Lovibond, 1995). In the present sample, Cronbach's alpha values were .91, .85, and .87 for the Depression, Anxiety, and Stress subscales, respectively.

#### Adverse childhood experiences

The Adverse Childhood Experiences Scale (ACES; Felitti et al., 1998) was used to assess childhood adversity. Participants were asked to indicate on a binary scale (0 = “no,” 1 = “yes”) whether they had been exposed to 10 specific experiences before 18 years of age (e.g., physical abuse, sexual abuse, neglect). The ACES is predictive of a range of adverse mental health outcomes, such as substance use, depression, and suicide (Merrick et al., 2017).

### Data analysis

In total, 385 data entries were received, with 189 fully complete entries (e.g., all items required for the model). Data preparation and analysis were completed using *R* software (Version 4.2.0; R Core Team, 2022). Missing data were deemed missing completely at random (Little, 1988) and imputed using predictive mean matching with the *mice* package (Version 3.16.0; R Core Team, 2022). Multiple imputation with 25 imputed datasets was utilized twice to compare the data when imputing from all entries versus first removing participants who only provided data for sex and age (*n* = 71). Occupation, years of work experience, ethnicity, province of residence, educational attainment, and marital status were included as predictors in the multiple imputation in addition to the variables in our analysis. Imputed data were checked against observed data with strip plots, and algorithm convergence was checked by inspecting plotted trace lines. No major differences were identified across analyses. We present the results of the imputed sample after removing participants who only provided sex and age; please see the Supplementary Materials for the latent variable models produced using the complete case data set and fully imputed dataset.

Descriptive statistics were calculated to characterize the sample. SEM was performed using the *runMI* function from the *semTools* package (Version 0.5–6; Jorgensen et al., 2022), which is a multiple imputation implementation of the *lavaan* package (Rosseel, 2012) and generates pooled parameter estimates and standard errors using Rubin's rule (Rubin, 1987). We performed Mardia's (1970) test for multivariate normality implemented in the *MVN* package (Korkmaz et al., 2014), which indicated significant multivariate skewness and kurtosis. Therefore, the robust maximum likelihood (MLM) estimator (i.e., robust standard errors and a Satorra–Bentler scaled test statistic) was used to appropriately handle multivariate nonnormality (Rosseel, 2012). Mahalanobis distance indicated one multivariate outlier, which was removed.

Two latent variables were constructed for the model: (a) MI and (b) PTSD. The MI latent variable included the MIOS Shame subscale, Trust Violation subscale, and single functional impairment item. The PTSD latent variable included the four PCL‐5 subscales (i.e., Intrusions, Avoidance, Negative Alterations in Cognitions and Mood, and Alterations in Arousal and Reactivity) and the MDI Depersonalization and Derealization subscales. The DASS‐21 subscales, ACES, age, and sex were entered as covariates for MI and PTSD, and MI was additionally included as a predictor of PTSD. The following conventions were used to evaluate model fit: comparative fit index (CFI) and Tucker–Lewis Index (TLI) values of .95 or higher, a standardized root mean squared residual (SRMR) less than .08, and a root mean square error of approximation (RMSEA) less than .06.

A follow‐up model was constructed to test the sensitivity of the original model. The follow‐up model was identical to the original model except that the PTSD latent variable included only the PCL‐5 subscales. The same covariates were entered in the follow‐up model, and the same conventions were used to evaluate model fit.

## RESULTS

### Original model

**FIGURE 1:**
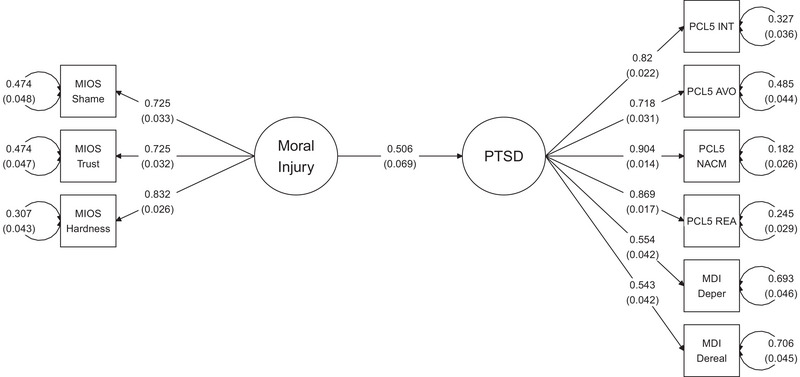
Structural equation model depicting associations between moral injury (MI) and posttraumatic stress disorder (PTSD) among public safety personnel *Note*: All values are standardized. Standard errors for residuals and covariances in parentheses. Covariates included sex, age, depressive symptoms, anxiety symptoms, stress‐related symptoms, and childhood adversity. The entire model, including covariates, explained 83.7% of the variance in the posttraumatic stress disorder (PTSD) latent variable. Figure constructed using the *tidySEM* package (Version 0.2.6; van Lissa et al., 2023). INT = intrusions; AVO = avoidance; NACM = negative alterations in cognition and mood; REA = alterations in arousal and reactivity; DPER = depersonalization; DREAL = derealization; MIOS = Moral Injury Outcomes Scale; PCL‐5 = PTSD Checklist for *DSM‐5*; MDI = Multiscale Dissociation Inventory.

The PTSD latent variable was regressed onto the MI latent variable to assess the impact of MI on PTSD (Figure 1). Fit indices for this model were as follows: robust CFI = .956, robust TLI = 1.0, robust RMSEA = .054, SRMR = .049. This model accounted for 83.7% of the variance in PTSD, *r* ^2^ = .837 *p* < .001. MI was strongly and significantly associated with PTSD, β = 0.506, *p* < .001, after controlling for age, sex, depression, anxiety, stress, and childhood adversity. There were significant positive effects of anxiety, β = 0.234, *p* < .001, and stress, β = 0.218, *p* = .002. Sex, age, depression, and childhood adversity were not significantly associated with PTSD, *p*s = .140–.789

### Follow‐up model

The PTSD latent variable, which did not include depersonalization or dissociation, was regressed onto the MI latent variable to compare results to the original model. Fit indices for this model were as follows: robust CFI = .901, robust TLI = 1.0, robust RMSEA = .080, SRMR = .180. This model accounted for 82.1% of the variance in PTSD, *r* ^2^ = .821 *p* < .001. MI was strongly and significantly associated with PTSD, β = 0.520, *p* < .001, after controlling for age, sex, depression, anxiety, stress, and childhood adversity. There were significant positive effects of anxiety, β = 0.205, *p* = .001, and stress, β = 0.225, *p* = .002. Sex, age, depression, and childhood adversity were not significantly associated with PTSD, *p*s = .166–.964

## DISCUSSION

The purpose of this study was to explore the association between MI and PTSD, including dissociative symptoms, in a sample of Canadian PSP, when controlling for the effects of sex, age, depression, anxiety, stress, and childhood adversity. The full model (including covariates) significantly accounted for a substantial proportion (83.7%) of the variance in PTSD symptom severity. MI was the strongest predictor among all covariates and was significantly and positively associated with PTSD beyond the contributions of sex, age, depression, anxiety, stress, and childhood adversity. Anxiety and stress were additionally found to be independently associated with PTSD. Our follow‐up model, which excluded depersonalization and derealization symptoms, further supported the strong association between MI and PTSD in this population. These results are consistent with other research pointing toward a strong association between MI and PTSD (Benfer et al., 2023; D'Alessandro‐Lowe et al., 2024; Easterbrook et al., 2022, 2023; Koenig et al., 2019; Levi‐Belz et al., 2020; Maguen et al., 2022; Roth et al., 2022, 2023). Our study extends existing research on the association between MI and PTSD among PSP by accounting for symptoms of depersonalization and derealization in the PTSD construct examined. Our model explained a comparable amount of variance in PTSD as was found among health care workers (87%; D'Alessandro‐Lowe et al., 2024), with a strong, positive, and significant association between MI and PTSD in both samples.

Further work is required to better understand the overlap and boundaries between MI and PTSD. The question of whether MI represents a distinct response necessitating its own diagnostic nosology remains a topic of debate in the field. Some scholars contend that the PTSD diagnostic criteria cannot fully capture MI and, thus, MI requires its own classification as a trauma‐related stressor (Farnsworth et al., 2017). Research with military populations suggests that some individuals present with a PTSD diagnosis without features of MI, some report MI without a PTSD diagnosis, and still others present with features of both PTSD and MI (Koenig et al., 2020, 2019). Moreover, MI is associated not only with PTSD but also with an array of other transdiagnostic psychopathology, including anxiety, depression, suicidality, and substance use (Hall et al., 2022), and PMIE exposure is associated with the development of a range of mental health disorders (Easterbrook et al., 2023). Alternatively, caution has been raised regarding the pathologizing of normative and adaptive responses to moral violations (Farnsworth et al., 2017). Indeed, dysphoric emotions and cognitions following PMIE exposure—namely, moral pain—may demonstrate an appropriate response to moral violations that can facilitate amend‐making. Whether MI represents a distinct traumatic response requiring separate diagnostic criteria and treatment is a question outside of the scope of the present paper but remains an important consideration for the field.

Our findings point toward the importance of considering MI when addressing PTSD among PSP. Symptoms stemming from shame‐related and trust violation–related MI, along with MI‐related functional impairment, should be acknowledged as relevant when considering PTSD among PSP. Individuals who work in public safety will continue to face PTEs and PMIEs due to the nature of their work, and, thus, they may benefit from support that addresses exposure to repeated PMIEs (e.g., having to make life‐and‐death decisions, witnessing human suffering; Easterbrook et al., 2022; Lade et al., 2023; Lentz et al., 2021; Phelps et al., 2023; Rodrigues et al., 2023; Smith‐Macdonald et al., 2021). Notably, existing evidence‐based treatments for PTSD, such as prolonged exposure and cognitive processing therapy, may not adequately target features consistent with MI due to the life‐threat and fear‐based focus of such existing treatments (Griffin et al., 2019). Future research is needed to best understand how existing therapeutic approaches to PTSD may be modified or augmented to account for experiences consistent with MI among PSP.

This study has several strengths, including the capture of responses from a range of PSP professions from across Canada and the meticulous data analytic strategy, which was used to compare the latent class SEM with a complete case analysis versus two types of multiply imputed datasets to address missing data. The study may be limited by its sample of PSP, which may not be representative of the PSP population in Canada (e.g., most resided in Ontario). Similarly, the sample scored low on clinical variables (e.g., PTSD, MI, dissociation, depression), which may have impacted the pattern of results. Future work should consider the association between MI and PTSD in a treatment‐seeking sample of PSP. Furthermore, although the MIOS, which was used to assess MI, was validated in military service members and acute care nurses, to our knowledge, its psychometric properties are yet to be determined among PSP. Finally, despite using the gold‐standard self‐report questionnaire for PTSD symptoms, the PCL‐5, we cannot claim that PSP participants in this study had a diagnosis of PTSD or its dissociative subtype given that Criterion A traumatic events were not measured nor were clinical interviews administered. PTSD symptoms are expected in the immediate aftermath of trauma exposure but are not necessarily indicative of a PTSD diagnosis. Accordingly, it is important to acknowledge that our study measured PTSD symptoms, not a PTSD diagnosis per se. Relatedly, our study did not account for types of trauma exposure (e.g., life‐threatening vs. non–life‐threatening), which may result in different sequelae and may be impacted by sociodemographic factors, such as sex or gender. The results may also have been impacted by participants’ trauma histories, which the ACES may have not sufficiently captured. Future research should account for trauma type when exploring the associations between MI and PTSD and their impacts on PSP. Finally, our study sought to understand the unique association between MI and PTSD, above and beyond related covariates (i.e., sex, age, depression, anxiety, stress, childhood adversity), and future work may consider exploring pathways that account for the nuanced associations among multiple factors.

The purpose of this study was to explore the unique association between MI and PTSD symptoms among Canadian PSP. Through latent variable SEM, we found evidence for a significant, positive association between MI and PTSD above and beyond the impact of relevant covariates. When addressing PTSD symptoms among PSP, it is important to also consider MI, which may be highly associated with PTSD symptoms and may warrant additional treatment considerations.

## AUTHOR NOTE

This research was supported by a grant from the Canadian Institutes of Health Research (Grant No.: W12 179950 to Margaret C. McKinnon), the Homewood Health Centre (donation to the Homewood Research Institute), the federally funded Atlas Institute for Veterans and Families, and the Horne Family Memorial Fellowship in Posttraumatic Stress injury and Recovery. Margaret C. McKinnon is supported as the Homewood Chair in Mental Health and Trauma at McMaster University. Ruth Lanius is supported as the Harris‐Woodman Chair in Psyche and Soma at Western University of Ontario. Andrea M. D'Alessandro‐Lowe is supported by the Horne Family Memorial Fellowship in Posttraumatic Stress Injury and Recovery, a CIHR Doctoral Graduate Scholarship (No. 493412), and the Research Institute of St. Joseph's Hamilton Studentship Award.

## OPEN PRACTICES STATEMENT

The study reported in this article was not formally preregistered. Neither the data nor the materials have been made available on a permanent third‐party archive; requests for the data or materials should be sent via email to the corresponding author at mckinno@mcmaster.ca.

## Supporting information



Supplementary Materials
