# Supplementary material for: Exploring the association between moral injury and posttraumatic stress symptoms among Canadian public safety personnel
Source: J Trauma Stress. 2024 Dec 16;38(2):272–83. doi: 10.1002/jts.23122 (PMC11967315; doi:10.1002/jts.23122)
Supplement: Supplementary file 1 — Supplementary Materials [file JTS-38-272-s001.docx]

**Supplementary Materials**

Below we provide the results of our modelling when using a complete case of the data and when using multiple imputation on all 385 cases (i.e., including those who only provided demographics). Model fit and statistics are comparable across analyses.

**Moral Injury and PTSD – COMPLETE CASE (N=189)**

The PTSD latent variable was regressed onto the moral injury latent variable to assess the impact of moral injury on PTSD (Figure 1). Fit indices for this model were as follows: robust CFI = 0.954, robust TLI = 0.937, robust RMSEA = 0.076, SRMR = 0.034. This model accounted for 92.3% of the variance in PTSD (*r*^2^ = 0.923). Moral injury strongly and significantly predicted PTSD (β = 0.759, *p* < 0.0001) after controlling for age, sex, depression, anxiety, stress and childhood adversity. There was a significant effect of anxiety (β = 0.218, *p* = 0.002) on PTSD. Sex, age, depression, stress and childhood adversity did not significantly predict PTSD symptoms (*p’s* > .05).

**Moral Injury and PTSD – IMPUTED (N=385)**

The PTSD latent variable was regressed onto the moral injury latent variable to assess the impact of moral injury on PTSD (Figure 1). Fit indices for this model were as follows: robust CFI = 0.962, robust TLI = 1.00, robust RMSEA = 0.039, SRMR = 0.054. This model accounted for 81.5% of the variance in PTSD (*r*^2^ = 0.815 *p* < .0001). Moral injury strongly and significantly predicted PTSD (β = 0.523, *p* < 0.0001) after controlling for age, sex, depression, anxiety, stress and childhood adversity. There were significant effects of anxiety (β = 0.240, *p* = 0.0001) and stress (β = 0.203, *p* < 0.007). Sex, age, depression and childhood adversity did not significantly predict PTSD symptoms (*p’s* > .05).
